# WORKSHOP ON TEXTURE IN ELECTRONIC APPLICATIONS Gaithersburg, MD October 10–11, 2000

**DOI:** 10.6028/jres.106.026

**Published:** 2001-06-01

**Authors:** Mark D. Vaudin, Debra L. Kaiser

**Affiliations:** Ceramics Division, Materials Science and Engineering Laboratory, National Institute of Standards and Technology, Gaithersburg, MD 20899-8522

## 1. Introduction

Many components and devices in electronic systems are fabricated from materials that have a preferred crystallographic orientation or texture. The applications in which the texture of the material plays a key role in determining the properties and performance are broad: Al and Cu interconnects in integrated circuits, complex oxides in random access memory devices, and metallic alloys in magnetic recording media are but a few examples. Texture is established during the synthesis or post-synthesis heat treatment of a material and thus has a strong dependence upon processing history. Accurate measurement of texture is not simple, and a variety of tools and approaches are being actively developed and employed in texture studies. x-ray, neutron, and electron diffraction based techniques are practiced around the world at varying levels of complexity with regard to equipment and analysis methods. Despite the well-documented existence of these varied approaches, many reported texture measurements on electronic materials are based solely on the relative intensity of a conventional θ–2θ x-ray diffraction peak, which can yield inaccurate results [1,2]. This observation was made by one of the authors of this report after listening to presentations at major materials conferences over several years and led to the initiation of a workshop on the subject “Texture in Electronic Applications.”

## 2. Objectives

The primary goal of this workshop was to provide a forum for the discussion of critical issues relevant to texture and texture measurement. The major topics were:

- Production and control of texture in a variety of different device materials
- Different methods of texture measurement and conditions for which each method is applicable
- Effects of texture on properties and performance
- Effects of texture on processing of subsequent layers deposited upon a textured template
- Texture analysis procedures

A deliberate effort was made to engage participants involved in a broad array of materials, measurement techniques, and application areas in order to provide an opportunity for meaningful interchange and collective insight into the measurement needs of the texture community.

## 3. Format

The 2 day workshop was co-chaired by M. D. Vaudin (NIST), K. P. Rodbell (IBM T. J. Watson Research Center), G. R. Fox (Ramtron International Corp.), and D. L. Kaiser (NIST). A letter describing the workshop was mailed to over 140 people either engaged in or concerned with texture measurement; the mailing list included industrial, government, and academic institutions. In addition, an e-mail notice was sent to the attendees of the 2000 Ceramics Gordon Conference on microstructure and texture in ceramics. Professors were encouraged to send their graduate students. The workshop was featured in the NIST Ceramics Division Events Calendar on the web: <http://www.ceramics.nist.gov/events/texture/contact.htm>.

The technical program consisted of 16 invited and contributed oral presentations, a poster session, and a round table, wrap-up discussion. An informal social gathering was held on the evening of October 10th at a local restaurant to promote further interaction and exchange of ideas. Outlines of the oral presentations were posted on the workshop web site, <http://www.ceramics.nist.gov/events/texture/texture.htm>, which was regularly updated until 1 week before the workshop. This innovative idea allowed prospective attendees to determine whether the workshop would be covering their particular areas of interest.

## 4. Content of the Workshop

The number of registered attendees was 38 with the following profile: 12 industry, 13 academic, and 13 government institutions. An additional 10 to 20 unregistered NIST employees were present at various times during the workshop. Representatives from the following institutions registered:

Companies:

American Superconductor (1)
Bede Scientific, Inc. (1)
HKL Technology, Inc. (1)
Hypernex, Inc. (2)
IBM (4)
Agere Systems (1)
Ramtron International Corporation (1)
Seagate Technology (1)

Government laboratories:

Naval Research Laboratory—NRL (1)
NIST (10)
Oak Ridge National Laboratory—ORNL (2)

Universities:

Boston University (1)
Carnegie-Mellon University (2)
McGill University (2)
Michigan State University (1)
North Carolina State University (1)
Pennsylvania State University (2)
Purdue University (1)
Rutgers University (1)
Israel Institute of Technology (1)
University of Connecticut (1)

The workshop was opened by the chief of the NIST Ceramics Division, Dr. Stephen W. Freiman, who welcomed the attendees, described the organizational structure of NIST, and outlined possible ways for NIST to interact with the texture community.

The following oral presentations were given:

1. *Workshop Introduction and Overview* (Mark Vaudin, NIST)
2. *Quantitative Texture Analysis of Blanket Films and Interconnects* (Chris Kozaczek, Hypernex)
3. *New Tools for Texture Analysis* (Jerzy Szpunar, McGill Univ.)
4. *Texture in Plated Cu Thin Films* (Ken Rodbell, IBM Yorktown Heights)
5. *Texture Inheritance in Al(Cu) Interconnect Materials* (Conal Murray, IBM Yorktown Heights)
6. *GaN Texture Control* (Alma Wickenden, NRL)
7. *Submicron-Resolution Texture and Strain Determination Using X-Ray Microbeams* (John Budai, ORNL)
8. *Characterization of Crystallographic Texture in Thin Films by Electron Diffraction* (Bin Lu, Seagate Technology)
9. *Texture in Magnetic Recording Media* (Michael Toney, IBM Almaden)
10. *Texture Evolution during Templated Grain Growth of Electrical Ceramics* (Gary Messing, Penn. State Univ.)
11. *Texture Development in Barium Strontium Titanate Thin Films Grown by MOCVD* (Tom Shaw, IBM Yorktown Heights)
12. *Epitaxy of Perovskite PZT Films Grown under Hydrothermal Conditions* (Kate Mikulka-Bolen, Rutgers Univ.)
13. *Polydomain Architecture of Epitaxial Ferroelectric Films* (Pamir Alpay, Univ. of Connecticut)
14. *Domain Textures in Piezoelectrics* (Keith Bowman, Purdue Univ.)
15. *Texture Dependence of Ferroelectric PZT Thin Film Properties Critical to FRAM Performance* (Xiao-Hong Du, Ramtron Corporation)
16. *Quantification of Microtexture using the Automated Electron BackScatter Diffraction Technique in the Scanning Electron Microscope* (John Sutliff, HKL Technology)

The following posters were presented:

1. *In Situ Neutron Scattering Study of Textured Rare-Earth Carbide Growth* (Louis Santodonato, NIST)
2. *The Use of Texture Data for the Calculation of Elastic Properties* (Thomas Gnaupel-Herold, NIST)
3. *Texture in Ti/Al and Nb/Al Multilayer Thin Films: Role of Cu* (Wayne Archibald, Carnegie Mellon Univ.)
4. *Texture by the Kilometer* (Eliot Specht, ORNL)

The first two talks (Mark Vaudin* and Kris Kozaczek*) provided overviews of texture in materials from the perspectives of measurement technique and the quantification of texture by the use of the orientation distribution function (ODF). Jerzy Szpunar* described advances in the measurement of texture in thin films and gave a large number of examples from diverse materials systems. In the succeeding talks, texture studies were presented in a large number of materials systems, all falling under the general description of “Electronic Applications.” The systems are summarized in Table 1.

A number of the presenters (marked * in the text above and in Table 1) have agreed to allow their presentations to be posted on the internet. Their viewgraphs are available as Portable Document Format (PDF) files on: <http://www.ceramics.nist.gov/events/texture/speakers.htm>.

## 5. Discussion

The final afternoon of the workshop was devoted to a round table discussion, and approximately 20 workshop attendees were present. A large number of topics related to many aspects of texture measurement were covered. Immediate action items were identified and plans for the future were outlined. The highlights are summarized here.

It was felt that the attendance level at this workshop (the first of its kind) was encouraging, as was the diversity of the attendees among industry, academia and national labs. However, it is obviously desirable to reach a wider audience, for which increased publicity and the further development of contacts are necessary. Since the workshop was held, this task has become easier as the International Committee on Texture of Materials (ICOTOM) has established a web site: <http://www.texture-anisotropy.org/>. The consensus of the discussion group was that a proceedings was not an appropriate workshop output. Rather, it was favored that presenters would have the opportunity of making their viewgraphs available for downloading from the web as discussed above. The other formal output of the workshop is this report.

It was generally felt that the meeting had been successful in its stated objectives, and that another similar meeting should be held in about 18 months to assess progress in the field. At the suggestion of Kenneth Rodbell (IBM), a committee was set up to organize a symposium on Texture and Microstructure in Electronic and Magnetic Films to be held at the Spring 2002 meeting of the Materials Research Society in San Francisco. Jerzy Szpunar (McGill Univ.), John Sutliff (HKL Technology) and Mark Vaudin (NIST) volunteered to be on the organizing committee; subsequent to the workshop, David Field (Univ. of Washington) and Pat DeHaven (IBM) have also agreed to join the committee. One focus area for the symposium will be the synthesis of texture measurements with microstructure analysis (grain size/morphology).

This workshop focused on the materials used in electronic and magnetic applications, which are typically in thin film form on planar substrates and have axisymmetric and typically sharp texture (at most a few degrees in full-width-at-half-maximum, FWHM). There was some discussion on the feasibility of applying bulk texture measurement technology used for traditional metallurgical and geological systems to thin films. Since texture in these bulk materials is usually three dimensional and broader than in films, it was felt that these more mature texture measurements are not directly applicable to films.

The role that NIST could play in assisting workers in the field of texture measurements was discussed in detail. Two potential NIST deliverables were a texture standard and a “Recommended Practice Guide.” Each of these is discussed below.

The general consensus was that the community needs a thin film texture standard for measurement calibration purposes. It was suggested that an informal round robin would be a good starting point for establishing specimen and measurement parameters for the eventual production of such a standard. The following institutions made verbal commitments to participate: ORNL, IBM, HKL Technology, and McGill University; others will be solicited. NIST’s role in this process would be to coordinate the distribution of specimens, make measurements, and collect, collate and compare the results. Eliot Specht (ORNL) volunteered to provide a length of rolled and annealed Ni tape (as used in the rolling assisted biaxially textured substrates (RABiTS) project [3]) as a candidate specimen which could be cut into lengths and distributed to various laboratories. This specimen has the advantage that long lengths of tape with the same texture can be prepared; Eliot Specht has developed a means of monitoring the texture to assess consistency. A specimen of this nickel tape has been sent to NIST and has been evaluated for suitability. The texture is more complex than is typical in an electronic film and current thinking is that blanket thin film specimens with sharp axisymmetric texture may be more suitable. However, the challenge will be to find a source of such films, all with the same texture. In conversations preceding the workshop, Glen Fox (Ramtron Corporation) had indicated that Ramtron can supply Pb*_x_* Zr_1−_*_x_*TiO_3_ (PZT) films on Pt/Si substrates with very consistent texture in both the Pt and PZT. In particular, the Pt films appear to be good candidate test specimens for the round robin studies and will be evaluated for suitability by NIST.

Recently, NIST has begun publishing “Recommended Practice Guides” on specific measurements, and there was discussion at the workshop on whether such a guide would be appropriate in the area of texture measurement. It was stated that many people are interested in texture but since “the science of texture measurement” is not well developed, there is currently no single “best practice.” Some of the issues raised by the participants were accuracy, procedures for correcting measured diffraction intensities, and selection of descriptors (pole figures, ODFs) and coordinate systems (Euler angles, direction cosines) for the various texture measurement techniques. Further discussions since the workshop have clarified the scope and target audience of a “Recommended Practice Guide.” They are aimed at the non-expert user, typically in an industrial setting. The emphasis is on obtaining data that is adequately accurate to resolve a particular technical question, and also on avoiding the pitfalls that beset any measurement. In this context, it is clear that NIST is in a position to offer suitable useful guidance regarding the use of conventional θ–2θ diffractometers to measure axisymmetric texture.

## 6. Conclusions

The workshop presented an excellent forum for discussion of a broad spectrum of texture-related issues of interest to academic, government and industrial participants. The need for a thin film texture standard was expressed and an informal round robin coordinated by NIST will be started this year as a first step in the standards development process. In addition, NIST will start working on a “Recommended Practice Guide” on texture measurement by conventional diffractometers. Finally, the group decided to hold a symposium on Texture and Microstructure in Electronic and Magnetic Films at the Materials Research Society Spring 2002 meeting.

## Figures and Tables

**Table 1:** Materials systems

Materials system	Application	Presenters
Cu and Al-5% Cu	Integrated circuit interconnects	Kenneth Rodbell* Conal Murray*
CoCrPt(B/Ta)	Magnetic recording media	Bin Lu Michael Toney*
Ti alloys	Turbines	John Sutliff
Ba*_x_* Sr_1−_*_x_* TiO_3_	Dynamic random access memories	Tom Shaw
Pb*_x_* Zr_1−_*_x_* TiO_3_	Transducers	Kate Mikulka-Bolen Keith Bowman
Pb*_x_* Zr_1−_*_x_* TiO_3_	Non-volatile random access memories	Xiao-Hong Du Pamir Alpay*
GaN	Lasers	Alma Wickenden
Pb(Mg_1/3_Nb_2/3_)O_3_ −35%PbTiO_3_ Sr*_x_* Ba_1−_*_x_* Nb_2_O_6_	Actuators	Gary Messing
High *T*_c_ superconductors	Power transmission	John Budai Eliot Specht
Rare earth carbides	Neutron diffraction research	Louis Santodonato
Metallic multilayers	Semiconductor metallization	Wayne Archibald
